# The Influence of Solar Ageing on the Compositions of Epoxy Resin with Natural Polyphenol Quercetin

**DOI:** 10.3390/ma17071592

**Published:** 2024-03-30

**Authors:** Malgorzata Latos-Brozio, Leszek Czechowski, Anna Masek

**Affiliations:** 1 Institute of Polymer and Dye Technology, Faculty of Chemistry, Lodz University of Technology, Stefanowskiego 16, 90-537 Lodz, Poland; 2 Department of Strength of Materials, Faculty of Mechanical Engineering, Lodz University of Technology, Stefanowskiego 1/15, 90-537 Lodz, Poland; leszek.czechowski@p.lodz.pl

**Keywords:** natural polyphenol, quercetin, epoxy resin, solar ageing

## Abstract

Epoxy resin compositions are used in modern railways, replacing other materials. However, epoxy composites in public transport are subject to many requirements, including that they should be flame retardant and resistant to weather conditions. The aim of the research was to analyse the resistance to solar ageing of epoxy resin composites containing flame retardants and the addition of the natural stabilising substance—quercetin. The homogeneity of the samples (optical microscopy and FTIR spectroscopy) and their thermal stability (TGA thermogravimetry) were analysed. The T5 temperature, which is the initial temperature of thermal decomposition of the samples, was 7 °C higher for the epoxy resin containing quercetin, so the material with polyphenol was characterised by better thermal resistance. Changes in material properties (hardness, surface energy, carbonyl index, colour) after 800 h solar ageing were investigated. The tensile tests on materials were executed for three different directions before and after ageing effect. The samples showed good resistance to degradation factors, i.e., they retained the functional properties (hardness and mechanical properties). However, analysis of carbonyl indices and surface energies showed that changes appeared in the composites after solar ageing, suggesting the beginning of material degradation. An approximately 3-fold increase in the polar component in epoxy resin compositions (from approximately 3 mN/m to approximately 11 mN/m) is associated with an increase in their hydrophilicity and the progress of ageing of the materials’ surface. The obtained results are an introduction to further research on the long-term degradation processes of epoxy resins with plant stabilisers.

## 1. Introduction

Epoxy resins are commonly utilised in different applications, such as coatings, adhesives, modelling compounds, high-performance composites, impregnation materials, insulating materials, encapsulating as well as packaging materials for electronic devices [1], deployable structures [2], and origami-inspired foldable composites [3]. Modern railways are an important sector in which materials based on epoxy resins are used. Epoxy compositions, due to their appropriate mechanical properties, have replaced metal parts of seats in trains [4,5]. However, materials used in this sector are subject to requirements regarding flammability [6] and, moreover, they should be resistant to weather conditions. The wide-scale industrial use of epoxy resins is due to their excellent properties: dimensional and chemical stability, fatigue strength, high specific stiffness, scratch resistance, and electrical resistance, as well as simple manufacturing and favourable costs [5,7]. Epoxy resins belong to the group of monomers and oligomers from which thermosetting materials with high chemical and physical resistance can be produced [8]. The structure of all epoxy resins contains a reactive epoxy ring (oxirane), which is responsible for the resin’s ability to cross-link. Epoxy rings have affinity for both nucleophilic and electrophilic groups and molecules, allowing resins to be cross-linked by various routes and mechanisms [9].

Polymer materials during exploitation, especially outdoors, are exposed to external factors causing degradation. One of the causes of ageing of polymers is solar ageing, related to the influences of solar radiation and increased temperature. Free radical production, which is the first stage of the degradation process, and the warming of the material compared to the ambient temperature are two effects of solar radiation that lead to radiation-related degradation of polymeric compositions [10]. Epoxy materials require the addition of stabilisers due to their low resistance to thermal, UV, and natural ageing [11]. These polymeric materials can be stabilised with both synthetic and natural-origin additives [11,12,13].

Natural compounds described in literature reports as stabilisers of polymeric materials include carotenoids, polyphenolic compounds, and phenolic polymers. The mechanism of the stabilising effect of polyphenol compounds and phenolic polymers is based on their participation in the transfer of hydrogen atoms and the chelation of transition metal ions in reactions with peroxide and alkoxy radicals and transition metals [14,15,16]. Quercetin is proposed as one of the polyphenols that can act as a polymer stabiliser. Quercetin (3,3′,4′,5,7-pentahydroxyflavone) is commonly found in vegetables and fruits [17]. Polyphenol has demonstrated several valuable health-promoting actions, including antioxidant, anti-UV radiation, antibacterial, as well as anticancer effects [18,19,20,21,22]. The literature reports concern the stabilisation of polyolefins [23], biodegradable polymers [24,25,26,27], poly(vinyl alcohol) [28], and elastomers [29,30] using quercetin. Few publications indicate the possibility of using quercetin to protect epoxy resin compositions against solar ageing. According to literature reports [31], composites were produced consisting of epoxy resin with flame retardants, glass fibres, potato starch, and quercetin. The starch biofiller was mixed with quercetin, and then this homogeneous powder was poured onto a composite consisting of a system of alternating layers of epoxy resin and glass fabric. Both natural substances prevented the hardening process of epoxy composites, which may occur during solar ageing [31].

The aim of this research is to analyse the stabilising effect of quercetin during solar ageing in composites based on commercial epoxy resins with flame retardants and glass fibres, specifically in materials dedicated for railways. Epoxy resin samples themselves are brittle, so it is necessary to make a composition with glass fibres to meet the properties suitable for railway seats. Glass fibres were used as a reinforcing filler of the sample, which should not affect the ageing process. According to the literature, the additive of glass fibre did not influence the behaviour of epoxy resin materials towards oxidative degradation [32]. Compositions of epoxy resin and glass fibres are typical materials used in railways, while natural additives such as quercetin were intended to act as pro-ecological stabilisers. Train carriages and their equipment are exposed to unfavourable effects of climatic factors, including solar radiation, which is why polymer materials (including seat elements) require protection against loss of properties and colour, yellowing, etc. The solar ageing time used in the research, 800 h, is approximately 1 month, to better approximate the effects on a longer time scale, e.g., a year. This work is an extension and continuation of the research published in one of our co-authored works [31] on epoxy composites with potential applications in the railway sector. Moreover, this research is an introduction to future work on the stabilisation of epoxy composites with natural substances during the long-term ageing process.

## 2. Materials and Methods

### 2.1. Preparation Method of Epoxy Resin Composites with Polyphenol (Quercetin)

Epoxy resin with flame retardants (tradename NEMresin 1011; New Era Materials, Modlniczka, Poland) was the basis for the production of polymeric materials. According to the manufacturer, the composition of the resin and flame retardant mixture was as follows: approx. 73 wt.% pure epoxy resin, <18 wt.% poly(ammonium phosphate), and approx. 9 wt.% graphite. Natural polyphenol quercetin (quercetin hydrate ≥ 95%, Sigma Aldrich, Munich, Germany) was introduced to the base resin blend in an amount of 2 phr (phr—parts per hundred resin). The epoxy resin was blended with polyphenols, and the powder was homogenised in a porcelain mortar and sifted through a sieve. The compositions consisted of alternating layers of epoxy resin and fabric made of fibreglass (RXT 350 g/cm^2^, Rymatex, Rymanów, Poland). During production of the samples, three layers of fibreglass fabric and three layers of resin were used, while maintaining the amount of resin at 450 g/cm^2^. Silicone foil was placed on the steel mould and a layer of fibreglass fabric was applied, followed by the blend of resin and quercetin (three alternating layers). Using a hydraulic press equipped with heating, plate-shaped samples were made. The process was two-stage. The first stage of the process consisted of plasticising the materials at 90 °C for 5 min. Subsequently, the materials were formed under the following conditions: 130 °C, 1 MPa, 8 min. The plate-like samples obtained in the above manner were hardened in a laboratory dryer (136 °C, 45 min).

### 2.2. Controlled Ageing in a Solar Chamber

For controlled solar ageing, the Atlas SC 340 MHG Solar Simulator (AMETEK Inc., Berwyn, IL, USA) containing a 2500 W MHG lamp was utilised. A specific range of IR, Vis and UV (solar) radiation was given by a halogen lamp with rare earth elements. The solar ageing conditions were as follows: time 800 h, temperature 70 °C, radiation intensity 1200 W/m^2^ (at 100% lamp power).

### 2.3. Optical Microscopy

The photos before and after solar ageing were taken with a Leica MZ6 stereoscopic microscope (Heerbrugg, Switzerland) utilising a ring light to illuminate the epoxy resin compositions. Software MultiScan 8.0 (CSS, Warsaw, Poland) was used to process and analyse the photos. The magnification of the photos was 130×.

### 2.4. Thermogravimetric Analysis (TGA) of Epoxy Resin Compositions

Thermogravimetric analysis was performed using a Mettler Toledo TGA/DSC 1 STARe device containing a GC10 gas controller (Greifensee, Switzerland). The measurement was carried out in the synthetic air oxidising atmosphere in the temperature range 25–1000 °C, rate of heating 20 °C/min, and flow of synthetic air 60 cm^3^/min. The test samples were placed in crucibles made of alumina.

### 2.5. Investigation of Surface Free Energy of Epoxy Compositions

The analysis of the surface free energy of epoxy compositions was done utilising an OEC 15EC goniometer (DataPhysics Instruments GmbH, Filderstadt, Germany) based on the determination of contact angles of three liquids, such as distilled water, ethylene glycol, and diiodomethane. The liquids were characterised by various polarities. The value of surface free energy was computed in the SCA 20 program using the Owens, Wendt, Rabel, and Kaelble (OWRK) method. Disperse and polar parts of surface energy as well as surface tension were combined by forming the sum of both parts, according to Equations (1) and (2):

(1) $$\sigma_{l}=\sigma_{l}^{d}+\sigma_{l}^{p}$$

(2) $$\sigma_{S}=\sigma_{S}^{d}+\sigma_{S}^{p}$$

where $\sigma_{l}^{d}$ and $\sigma_{l}^{p}$ represent polar and disperse parts of the liquid, and $\sigma_{s}^{d}$ and $\sigma_{s}^{p}$ correspond to the appropriate contributions of the solid.

### 2.6. Determination of Carbonyl Indices (CI) Based on Fourier-Transform Infrared Spectroscopy (FTIR)

The spectra of the epoxy compositions were recorded using a Thermo Scientific Nicolet 6700 FT-IR Spectrometer (Thermo Fisher Scientific, Waltham, MA, USA) equipped with a diamond accessory (Smart Orbit ATR). After placing the tested materials at the output of IR beams, FTIR spectra were recorded (wavenumber range 4000–400 cm^−1^, 64 scans, absorption mode). Analysis of vibrational FTIR spectra allows the examination of functional groups with which IR radiation interacts.

The carbonyl index (CI) was computed from the FTIR spectra according to Equation (3). CI corresponds to the number of carbonyl groups appearing during solar ageing of epoxy samples.

(3) $$CI=\frac{I_{C=O}}{I_{C-H}}$$

where:

I_C=O_ represents peak intensity characteristic of C=O carbonyl groups (~1700 cm^−1^) [-], and
I_C-H_ represents peak intensity characteristic of C-H aliphatic carbon (~2800 cm^−1^) [-].

### 2.7. Hardnesses of Epoxy Compositions on the Shore C and Shore D Scale

The hardness of materials made on the basis of epoxy resin was determined using Shore type C and D durometers (Zwick/Roell, Herefordshire, UK). The results are determined by measuring the depth of the indentation in the polymer sample created under the influence of a given force acting on a standardised pressure foot.

### 2.8. Determination of the Change in Colour Parameters of Epoxy Composites after Solar Ageing

Colour tests were carried out on aged and unaged samples of epoxy compositions. A CM-3600d spectrophotometer (Konica Minolta Sensing, Osaka, Japan) was utilised for colour assessment. The results of the study were the colour presented in the CIE-Lab space and the colour in the three-coordinate system marked as L, a, and b. The L parameter stands for lightness, and its maximum value of 100 indicates a perfectly reflective diffuser, while the minimum value of zero corresponds to the black colour. Parameter a denotes the red-green axis, and b corresponds to the yellow-blue axis. The a and b axes do not have characteristic numerical limits. Based on Equation (4), the colour change coefficient dE × ab was computed.

(4) $$dE\times ab=\sqrt{{(∆a}^{2})+\left({∆b}^{2}\right)+\left({∆L}^{2}\right)}$$

### 2.9. Uniaxial Tensile Test of Aged and Unaged Epoxy Compositions

**Figure 1:**
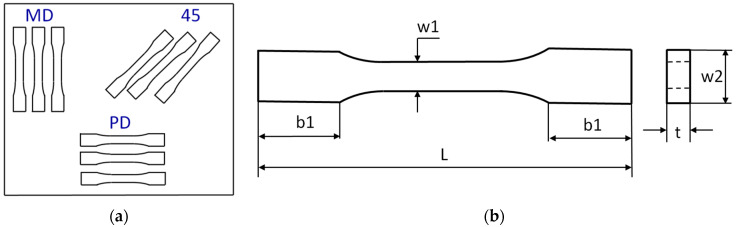
Arrangement of cut samples (**a**) and simple sketch (**b**).

Tensile tests were based on the UNE EN ISO 527-1:2020-01 standard [33]. The speed of moveable traverse was set at 2 mm/min. As effects of tests, the force vs. strains of sample charts were registered. The samples were taken basing on the scheme shown in Figure 1a, where MD is main direction, PD is perpendicular direction, and 45 means an angle orientated to the plate edges. The dimensions of all samples were taken into consideration: L = 170 mm, b1 = 40 mm, w1 = 10 mm, w2 = 20 mm, and mean thickness is about t = 3 mm (Figure 1b). Young’s moduli of samples were determined by means of a gauge with length 50 mm.

## 3. Results and Discussion

**Figure 2:**
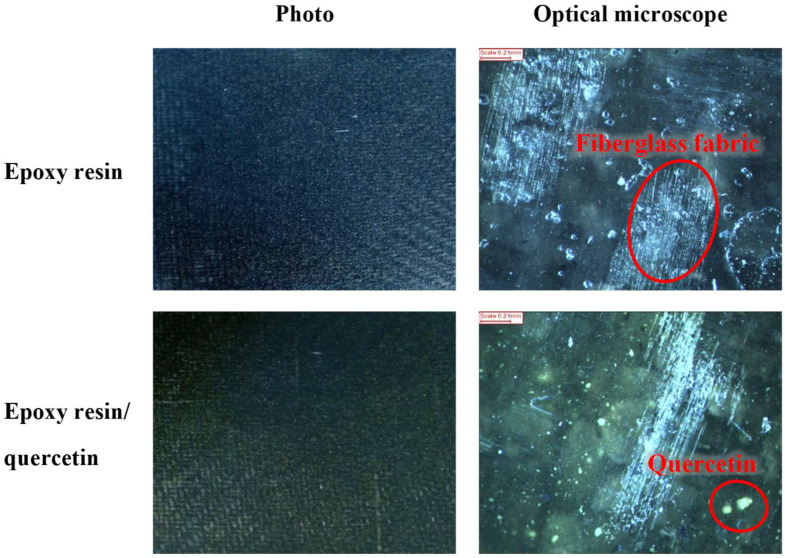
Photographs and microscopic photographs (130× magnification) of a reference sample of resin and composition with quercetin.

The first step of the study was to assess the homogeneity of samples with the natural additive. Figure 2 shows photographic and microscopic photos of the reference resin composition and the sample with quercetin. Based on visual assessment (photographs) and microscopic images (magnification 130×), it was found that the materials are homogeneous. Visible in all samples were glass fibres and flame-retardant mixture, and in the material with quercetin, additionally, characteristic yellow spots of polyphenol. The distribution of flame retardants (including graphite) and quercetin indicated that the samples were homogeneous. No larger agglomerates of additives were found. To fully assess the homogeneity of samples, in the future, microscopic examinations could be extended by taking photos on microscopes with higher magnifications or taking photos with a scanning electron microscope (SEM).

**Figure 3:**
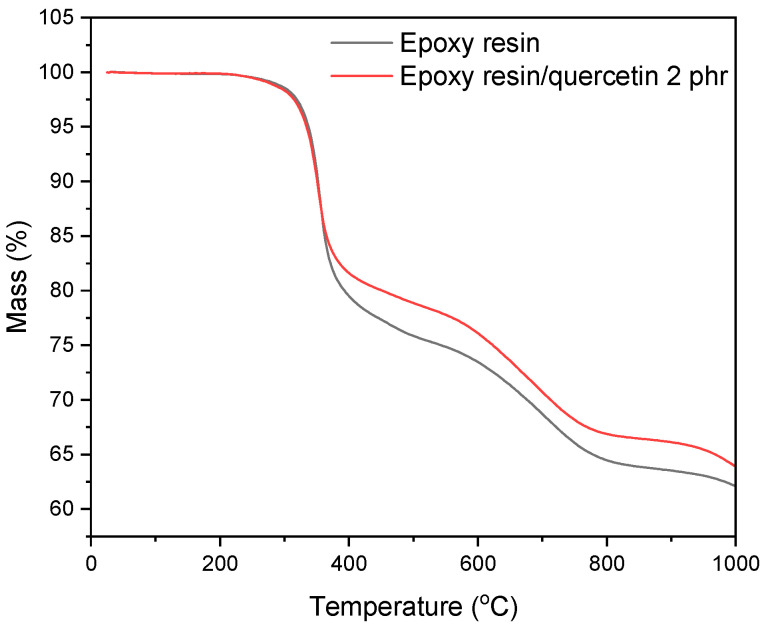
Thermogravimetric analysis (TGA) of epoxy resin compositions.

In the next stage, thermal analysis of the samples (Figure 3) was performed. The TGA curves showed that the decomposition of the reference resin composition and the quercetin material was two-step. The polyphenol-containing sample was characterised by a higher thermal resistance compared to the standard. The T5 temperature, which is generally considered to be the initial thermal decomposition temperature of the sample, was 327 °C for the epoxy resin composition and 334 °C for the epoxy resin/quercetin. The residue was 62.1 wt.% for the standard material and 63.9 wt.% for the composition with quercetin. The obtained research results correspond with other scientific reports. According to the literature, quercetin was tested as a thermal stabiliser of polypropylene (PP), and the addition of this polyphenol upgraded the resistance of the polyolefin to thermal degradation and increased the onset of decomposition temperature by approximately 35 °C [34]. In the publication [31] on epoxy resin/glass fibre/starch/quercetin compounds, the authors found that the addition of 2 phr of flavonoid led to an increase in T5 temperature of 11 °C compared to the epoxy resin/glass fibre/starch sample, which confirmed the stabilising effect of quercetin.

**Figure 4:**
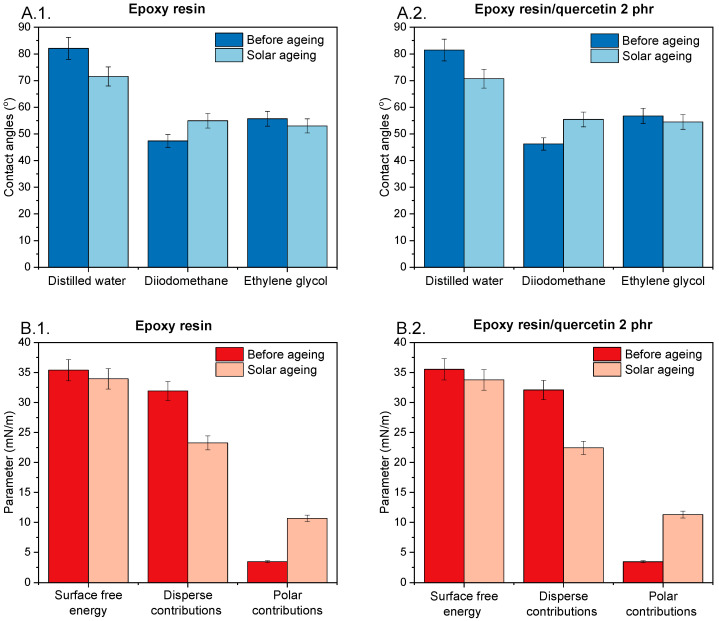
Change in the value of contact angles (**A.1.**,**A.2.**) and surface energy parameters (**B.1.**,**B.2.**) after solar ageing.

Figure 4 summarises the analysis of contact angles (Figure 4(A.1.,A.2.)) and surface energy parameters (Figure 4(B.1.,B.2.)) of unaged and aged epoxy resin samples. The average values of the surface free energy parameters of the reference sample (surface free energy 35.39 mNm; dispersion component 31.92 mNm; polar component 3.48 mNm) and samples with quercetin (surface energy 35.54 mNm; dispersion component 32.08 mNm; polar component 3.45 mNm) before ageing were identical. This means that the addition of 2 phr of quercetin had no significant effect on the surface properties of the composition. Controlled ageing in a solar chamber did not cause clear changes in the value of total surface free energy. However, the share of individual components changed significantly, and an increase in the value of the polar component and a decrease in the value of the dispersion component of surface free energy were noted. Higher values of the polar component in epoxy resin compositions correspond to the increase in the hydrophilic nature of the samples related to ageing and degradation occurring on the surface of the tested materials. Moreover, functional groups are formed on the surface of epoxy compositions related to the degradation processes of materials accompanying ageing in the solar chamber. Ageing results in the appearance of specific functional groups such as hydroxyl groups. The generation of OH groups on the resin surface resulted in an increase in the polar component and corresponded to the initiation of the degradation reaction of the surface of epoxy resin materials. An increase in the polar component of samples after solar ageing was also described for the compositions of epoxy resin containing the biofiller starch with flavonoid quercetin [31], as well as for composites of epoxy resin with another polyphenol—naringenin [35].

**Figure 5:**
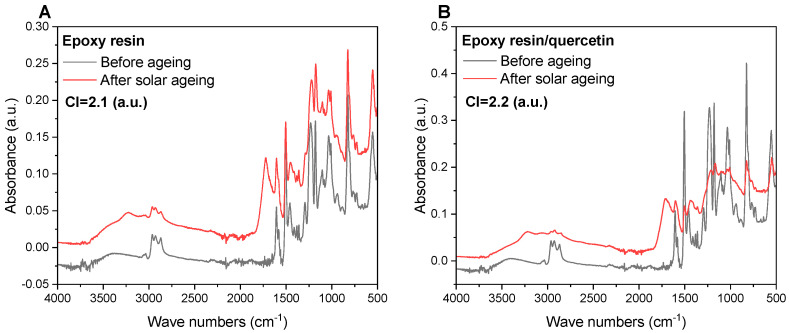
FTIR spectra of epoxy resin (**A**) and epoxy resin/quercetin (**B**) before and after solar ageing (CI symbolises carbonyl index).

During degradation (e.g., ageing in a solar chamber), changes appear in the chemical structure of polymers, which can be noticed by analysing the FTIR spectra (Figure 5). These changes may contribute to the detachment of functional substituents, as well as changes at the level of bonds-cracks of C–C or C–H bonds in the main chain and the formation of carbonyl, hydroxyl, and peroxide groups. The carbonyl index (CI) is considered a measure of the oxidation and degradation progress of polymeric materials, as it determines the concentration of the carbonyl groups (acids, aldehydes, and ketones) in the sample. The index shows the course of the degradation process, where the first stage is the oxidation of the polymer chain to carbonyl groups, which are crucial during oxidation (they decompose into CO_2_ and H_2_O). The occurrence of additional OH hydroxyl groups (present in the range of 3500–3000 cm^−1^) was found in FTIR spectra after solar ageing (Figure 5A,B). The OH groups change the polarity of the compositions, including surface free energy parameters. In addition, the presence of ketone groups was noted at the wave number of about 1700 cm^−1^, enabling the computation of carbonyl indices. The carbonyl index (CI) values were, respectively, 2.1 (a.u.) for the reference resin and 2.2 (a.u.) for the quercetin sample. The results suggest that the degradation progress was comparable in both materials. It seems that the addition of quercetin (2 phr) did not have a clear effect on the kinetics of the ageing process under the given conditions. In the case of analogous compositions based on epoxy resin, another polyphenol naringenin showed a stabilising effect on the materials (lower carbonyl indices compared to the reference sample) [35]. A detailed analysis of the bands in the FTIR spectra was described in previous thematically related publications by the authors [31,35].

**Figure 6:**
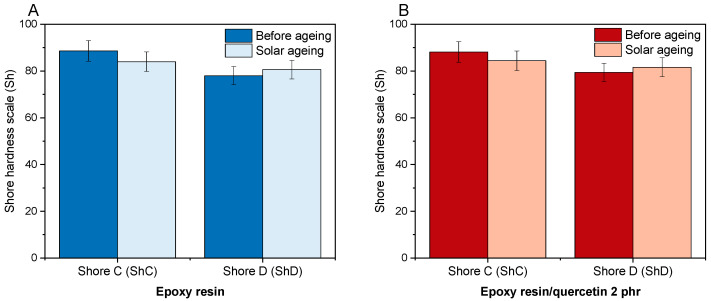
Change in the hardness value of epoxy resin (**A**) and epoxy resin/quercetin (**B**) before and after solar ageing.

Figure 6 shows the difference in the hardness value of the polymer compositions after solar ageing. Before ageing, the average hardness of the reference resin was 88.6 ShC and 78.0 ShD (Figure 6A). Introducing quercetin into the resin at a concentration of 2 phr did not change the hardness values measured on either scale (mean value difference ± 1) (Figure 6B). According to the Shore D scale, materials with a hardness above 60 ShD are considered extra hard. All epoxy resin samples both before and after ageing had hardness values above 78 ShD, so they were extra-hard materials. Moreover, ageing of materials in a solar chamber did not cause clear changes in the hardness of either composition. This proved that the material did not lose its resistance to local plastic deformation of the materials and that the functional properties have not been deteriorated during solar ageing. A comparable lack of negative impact of solar ageing on the hardness of polymeric samples was described for the compositions epoxy resin/glass fibres/starch/quercetin [31] and epoxy resin/glass fibres/naringenin [35].

For the duration of using polymeric materials, the colour change may be influenced by ageing factors, including UV radiation and increased temperature. Table 1 summarises the results of the testing of the colour changes of the epoxy samples after solar ageing. The colour change coefficient was calculated based on the lightness parameter L and the parameters of the a-axis (red-green axis) and the b-axis (yellow-blue axis) according to Equation (4). The colour change coefficient dE × ab for the epoxy resin was 2.25 (-), which statistically means that the difference in the colour of aged and unaged materials is noticed by an inexperienced observer (the range 2 < dE × ab < 3.5 means that an inexperienced observer will notice a colour change). The sample with quercetin was characterised by a higher dE × ab coefficient of 4.96 (-). In the range of 3.5 < dE × ab < 5, the observer perceives a significant difference in the colours. The more pronounced colour change of the material with quercetin was caused by the oxidation process of the flavonoid. Analogous results of greater material colour change due to flavonoid oxidation were found in a report on epoxy resin/glass fibre/naringenin composites. Moreover, as the concentration of polyphenol naringenin in the samples increased, a greater difference in the colour of the compounds was confirmed [35].

**Figure 7:**
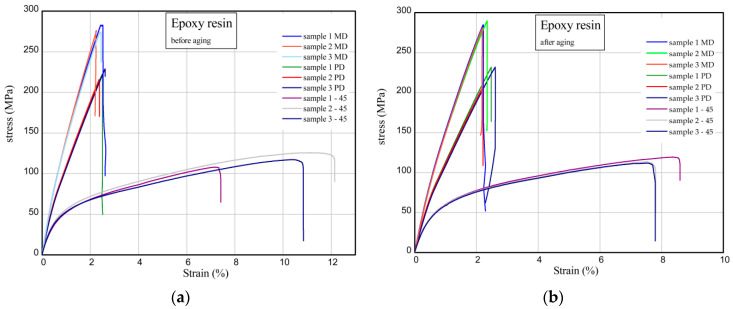
Curves of tension for reference epoxy resin before (**a**) and after (**b**) solar ageing.

**Figure 8:**
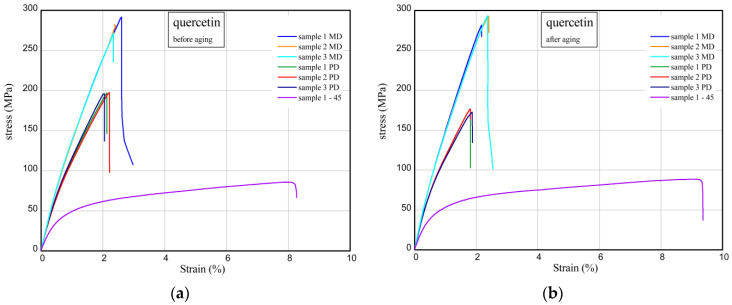
Curves of tension for epoxy resin/quercetin samples before ageing (**a**) and after solar ageing (**b**).

The results of tensile measurements of unaged and solar aged samples are shown in Figure 7 and Figure 8. The tensile characteristics were analysed for three directions (MD, PD, 45). Figure 7 shows the strain (in %) vs. normal stress (in MPa) for reference material (denotation: epoxy resin). Maximum stress and Young’s modulus for MD compositions were achieved at 278–285 MPa and Young’s modulus at 17.3–18.4 GPa. In the case of PD samples, Young’s moduli are smaller by 14–21% in comparison with Young’s moduli of MD samples. For samples taken at an angle of 45, mechanical parameters are meaningfully lower (over two times, at most). As a result of solar treatment, the strength of material is slightly higher (See Figure 7b). Typical parameters of the studied epoxy compositions are presented in Table 2 and Table 3. Considering the next charts (Figure 8), the addition of quercetin in composite effects a slight decrease in the stiffness as well as a light increase in maximum stress. The mechanical parameters of samples with quercetin after solar process are rather comparable. For analogous composites containing a biofiller (epoxy resin/glass fibres/starch/quercetin), generally, the presence of 15 phr of starch in the materials resulted in a slight decrease in strength and stiffness; however, the addition of 2 phr of quercetin reduced mechanical parameters before and after solar ageing [31].

## 4. Conclusions

The addition of quercetin increased the thermal stability of the epoxy resin/glass fibre materials. The tests confirmed that the samples retained their functional properties, including a hardness similar to the initial one, as well as mechanical properties. However, the study of FTIR spectra and surface energy parameters clearly showed that after solar ageing, changes appeared in the materials suggesting the beginning of sample degradation, including OH and ketone functional groups characteristic of ageing and an increase in the polar component. The longer experiment time probably allowed for the observation of further progress in the degradation of epoxy materials and the assessment of the effect of quercetin during long-term ageing. After ageing, the composition containing quercetin exhibited a more intense colour change, which was related to the oxidation processes of polyphenol.

## Figures and Tables

**Table 1:** Analysis of the change in colour of epoxy resin composition after solar ageing.

Parameter	dE × ab (-)	L* (-)	a* (-)	b* (-)
Epoxy resin				
Before ageing	-	35.05 ± 1.75	−0.40 ± 0.02	3.02 ± 0.15
Solar ageing	2.25 ± 0.11	13.25 ± 0.65	1.56 ± 0.07	2.37 ± 0.12
Epoxy resin/quercetin 2 phr				
Before ageing	-	35.12 ± 1.77	−4.23 ± 0.21	8.40 ± 0.42
Solar ageing	4.96 ± 0.25	33.31 ± 1.67	−0.33 ± 0.02	5.97 ± 0.30

**Table 2:** Main values of the Young’s modulus (in GPa).

Sample	Unaged			Solar Aged		
	MD	PD	45	MD	PD	45
Epoxy resin	17.30 ± 0.20	14.35 ± 0.40	9.36 ± 0.65	18.41 ± 0.24	14.76 ± 0.45	10.32 ± 0.18
Epoxy resin/quercetin 2 phr	16.28 ± 0.17	14.83 ± 0.47	8.54	17.86 ± 0.19	15.86 ± 0.19	9.41

**Table 3:** Main values of maximum stress (in MPa).

Sample	Unaged			Solar Aged		
	MD	PD	45	MD	PD	45
Epoxy resin	277.8 ± 4.6	223.8 ± 6.7	116.7 ± 8.9	284.7 ± 4.7	223.6 ± 14.2	114.8 ± 4.0
Epoxy resin/quercetin 2 phr	281.9 ± 10.0	196.8 ± 0.6	85.7	289.3 ± 6.6	175.2 ± 2.2	88.5

## Data Availability

Data are contained within the article.
